# The EPICure Study: Association between Hemodynamics and Lung Function at 11 Years after Extremely Preterm Birth

**DOI:** 10.1016/j.jpeds.2012.03.052

**Published:** 2012-10

**Authors:** Charlotte E. Bolton, Janet Stocks, Enid Hennessy, John R. Cockcroft, Joseph Fawke, Sooky Lum, Carmel M. McEniery, Ian B. Wilkinson, Neil Marlow

**Affiliations:** 1 National Institute for Health Research, Nottingham Respiratory Biomedical Research Unit, University of Nottingham, City Hospital, Nottingham, United Kingdom; 2 Portex Unit, Respiratory Medicine and Physiology, University College London, Institute of Child Health, London, United Kingdom; 3 Wolfson Institute, Queen Mary University of London, London, United Kingdom; 4 Wales Heart Research Institute, Cardiff University, Cardiff, United Kingdom; 5 School of Human Development, University of Nottingham, Nottingham, United Kingdom; 6 Clinical Pharmacology Unit, University of Cambridge, Cambridge, United Kingdom; 7 Institute for Women's Health, University College London, London, United Kingdom

**Keywords:** AIx, Augmentation index, BP, Blood pressure, BPD, Bronchopulmonary dysplasia, COPD, Chronic obstructive pulmonary disease, EP, Extremely preterm, FEF_25-75_, Forced mid-expiratory flow, FEV_1_, Forced expiratory volume in 1 second, FVC, Forced vital capacity, MAP, Mean arterial pressure

## Abstract

**Objective:**

To investigate the relationship between disturbed lung function and large-artery hemodynamics in school-age children born extremely preterm (EP) (at 25 completed weeks of gestation or less).

**Study design:**

This was a cross-sectional study of participants from the EPICure study, now aged 11 years (n = 66), and 86 age- and sex-matched term-born classmates. Spirometry parameters (including forced expiratory volume in 1 second), blood pressure, and augmentation index (AIx, a composite of arterial stiffness and global wave reflections) were measured.

**Results:**

Compared with their classmates, the EP children had significantly impaired lung function, particularly those with neonatal bronchopulmonary dysplasia. Peripheral blood pressure did not differ significantly between the 2 groups, but AIx values were on average 5% higher (95% CI, 2%-8%) in the preterm infants, remaining significant after adjustment for potential confounders. Neonatal bronchopulmonary dysplasia status was not related to AIx. Lung function and maternal smoking were independently associated with AIx; AIx increased by 2.7% per *z*-score reduction in baseline forced expiratory volume in 1 second and by 4.9% in those whose mothers smoked during pregnancy.

**Conclusion:**

The independent association between impaired lung function and cardiovascular physiology in early adolescence implies higher cardiovascular risk for children born EP, and suggests that prevention of chronic neonatal lung disease may be a priority in reducing later cardiovascular risk in preterm infants.

Alongside a number of traditional risk factors, disturbed lung function has emerged as a key independent risk factor for cardiovascular disease in adults.^1,2^ Recent work has identified the independent, indirect relationship between lung function and arterial stiffness, itself an independent cardiovascular predictor,^3^ in adults with chronic respiratory diseases, such as chronic obstructive pulmonary disease (COPD)^4,5^ and cystic fibrosis, as well as in an unselected population of adult males.^6,7^

Impaired lung function has repeatedly been demonstrated in children born very preterm, independent of chronic neonatal lung disease or bronchopulmonary dysplasia (BPD).^8^ Both structural and functional impairments in lung function appear to persist into adulthood.^9-11^ The implications of preterm birth are of increasing relevance to adult physicians, given improved survival,^12^ tracking of lung function throughout life,^13-15^ and concerns that the normal age-related decline in lung function may be accelerated in such individuals.^16^ There is growing concern regarding the long-term effects of adverse early life events, including fetal programming, along with altered prenatal and postnatal growth patterns, which have been implicated in systolic hypertension and cardiovascular disease.^17,18^

The EPICure study identified significantly reduced lung function but normal brachial blood pressure (BP) in 6-year-old children born at 25 weeks completed gestation or less in the United Kingdom and Ireland compared with national standards and classmates.^19,20^ Brachial BP traditionally has been used to assess hemodynamic status, because of its ease and ready accessibility. However, BP varies throughout the arterial tree due to variations in arterial compliance and augmentation by wave reflections; thus, peripheral BP measurements might mask altered central hemodynamics.^3^ Vascular properties are susceptible to change, promoting stiffness and increased wave reflection from the peripheries. Central hemodynamics were not measured in the children at 6 years of age in the EPICure study.

Several previous studies have explored the association of arterial stiffness and birth weight^21-24^; however, other studies have found inconsistent findings or have not demonstrated this association.^25,26^ Those studies focused on intrauterine growth restriction as opposed to prematurity. Central hemodynamics were measured at 11 years of age in the EPICure study. We have reported that the augmentation index (AIx), a composite vascular parameter of arterial stiffness and global peripheral wave reflection, is increased in children born extremely preterm (EP) compared with classmates.^3,27^ In younger individuals, AIx is more closely related to cardiovascular risk factors compared with more direct measures of large artery stiffening, such as aortic pulse wave velocity, the speed of pulsation down the aorta between the carotid and femoral pulsations, an independent predictor of cardiovascular disease in older adult subjects.^3,28^

Given the known impaired lung function in this population,^29^ we hypothesized that functional impairment would parallel altered central hemodynamics at age 11 years in children born EP. If so, then strategies to protect lung function during the neonatal period may have further important additional benefits for cardiovascular physiology and in limiting cardiovascular disease in later life.

## Methods

EPICure is a population-based study of all births between March and December 1995 in the United Kingdom and Republic of Ireland, born at 25 completed weeks of gestation or less (the data-sharing policy is available from www.epicure.ac.uk). Details of the cohort ascertainment and progress have been reported elsewhere.^19,20^ The current investigation was part of an extensive assessment of this cohort at age 11 years comprising neurocognitive, respiratory, and other outcomes.^29-33^ Measurements were performed in school by 1 of 3 specially trained pediatricians. Schools were asked to identify up to 3 classmates as potential comparisons, matched for age, sex, and ethnic origin, 1 of whom was selected at random.^29,30^ Classmates were excluded if born at <37 weeks gestational age, hospitalized for respiratory complaint or had sustained tuberculosis, pneumonia, or whooping cough. Asthma and atopy were not exclusion criteria. The study protocol was approved by the Southampton and South West Hampshire Research Ethics Committee. All parents gave written consent, and the children provided verbal agreement.

Parents were contacted before the assessment to verify that their child was well and free from any respiratory tract infections to ensure that measurements were obtained during a period of clinical stability. All bronchodilators and leukotriene antagonists were withheld on the day of testing.

### Standard Clinical Assessment

Weight was measured to the nearest 0.1 kg on a digital scale (BWB 600; Tanita, Tokyo, Japan), with the child wearing minimal clothing and barefoot. Height was recorded with the child barefoot to the nearest 0.1 cm using a Leicester stadiometer (Crawlea Medical, Birmingham, United Kingdom). Height and weight were converted to *z*-scores to adjust for sex and age.^34^ Questionnaires eliciting information on perinatal history, current health, therapies, and maternal smoking were completed by parents.

### Hemodynamic Assessment

The ability to include hemodynamic assessments in the protocol became available only during the latter part of the data collection period. After extensive training, a single pediatrician (J.F.) performed these measurements in children tested from September 2006 onward, at 1 hour after spirometric testing. After supine peripheral BP measurement (Omron, Kyoto, Japan) as the mean of 3 consecutive brachial artery readings, radial artery waveforms were recorded using a high-fidelity micromanometer (Millar Instruments, Houston, Texas). Pulse-wave analysis (Sphygmocor; AtCor Medical, Sydney, Australia) was used to generate a corresponding central waveform through a validated transfer function.^35^ The AIx is defined as the difference between the second and first systolic peaks, expressed as a percentage of pulse pressure.^27^ Pulse pressure amplification was calculated as the ratio of peripheral pulse pressure to central pulse pressure. Carotid-femoral (aortic) pulse wave velocity was measured as described previously,^27^ with results expressed as the mean of duplicate readings from traces reviewed and selected for quality by an independent experienced operator (C.M.) blinded to birth status.

### Lung Function Assessment

Baseline spirometry (Jaeger Masterscope V4.6; CareFusion, Wurzberg, Germany) was performed according to published guidelines.^29^ Consenting subjects were retested after administration of salbutamol 200 μg via a spacer device. A positive bronchodilator response was defined as a >12% increase in forced expiratory volume in 1 second (FEV_1_). All data were sent to the University College London, Institute of Child Health for centralized analysis and quality control by respiratory physiologists, blinded to the children's birth status.^29^ Results were expressed as *z*-scores to adjust for sex, age, and height.^36^

### Statistical Analyses

Data from lung function and hemodynamic assessments were combined with the main EPICure database for analysis. Forward stepwise regression was used to identify independent effects on AIx; predictors considered were standardized measures of lung function, sex, birth status, BPD (defined as receiving supplemental oxygen at 36 weeks postmenstrual age), maternal smoking during pregnancy, heart rate, mean arterial pressure (MAP), age, and height at time of test, in addition to their interactions with EP status. AIx values are based on unadjusted data. A *P* value <.05 was considered to indicate statistical significance. Analyses were performed using SPSS version 11.0 (SPSS Inc, Chicago, Illinois) and Stata release 10.1 (StataCorp, College Station, Texas).

## Results

Of the 307 EPICure survivors known to be alive at age 11 years, 11 were abroad, 20 refused participation in this study, and 57 were nonresponders. Valid spirometry data were obtained from 187 of the 219 children (85%) whose parents consented to the study, 66 (35%) of whom (including 45 [68%] with previous BPD) also underwent hemodynamic assessment. Of 170 classmate controls contacted, 161 produced valid spirometry data, of whom 86 (53%) also had data from hemodynamic studies. Characteristics of the study subjects are presented in Table I. The subgroup of EPICure children who underwent hemodynamic assessment was representative of the entire cohort tested in school^29^ with respect to neonatal history, respiratory morbidity, maternal characteristics, and lung function testing results (Table II; available at www.jpeds.com), but were slightly older, reflecting their evaluation toward the end of the recruitment period.

### Hemodynamic Assessment

Peripheral diastolic BP and MAP were marginally higher in the EP children compared with their classmates (mean difference, 2.1 mmHg [95% CI, −0.1 to 4.3 mmHg] and 2.1 mmHg [95% CI, −0.1 to 4.2 mmHg], respectively; both *P* = .06), whereas peripheral pulse pressure was −3.6 mmHg (95% CI, −6.6 to −0.6) lower (*P* < .05) (Table III; available at www.jpeds.com). AIx was significantly (*P* < .001) higher in the EP children (7% ± 10% vs 2% ± 9%: difference, 5% [95% CI, 2%-8%]) and remained so after adjustment for age, sex, MAP, and height (adjusted difference, 4% [95% CI, 0.4%-7%]). Only a minimal difference in AIx was detected between EP children with previous BPD and those without BPD unadjusted difference [BPD − no BPD], 1% [94% CI, −4 to 6]; adjusted difference, 2% (95% CI, −4% to 7%). No relationship between AIx and gestational age or birth weight was identified in the EP children. Pulse pressure amplification was lower in the EP children than their classmates, and aortic pulse wave velocity was similar in the 2 groups. The AIx was lower in boys than in girls in the EP group, but this sex difference was not evident in the classmates, and there was no significant interaction between sex and EP status (*P* = .50).

### Lung Function

Even after correction for height, age, and sex using standardized *z*-scores, children born EP had significantly worse baseline lung function (difference in *z*-scores: FEV_1_, −1.02 [95% CI, −1.37 to −0.66]; forced vital capacity [FVC], −0.49 [95% CI, −0.83 to −0.15]; FEV_1_/FVC ratio, −0.79 [95% CI, −1.13 to −0.44]; forced mid-expiratory flow [FEF_25-75_] −1.19 [95% CI, −1.57 to −0.81]) compared with their classmates, with these differences most marked in those with previous BPD (Table III). A positive bronchodilator response was observed more frequently in the EP children than in classmates (36% vs 9%), but postbronchodilator FEV_1_ was significantly lower in the EP children, indicating some degree of fixed airway obstruction. Current or previous (within 12 months) use of bronchodilators, inhaled corticosteroids, and leukotriene antagonists was more common in the EP children (data not shown).

### Relationship of Lung Function and Hemodynamic Parameters

In the EP children, AIx was inversely related to baseline lung function (FEV_1_ *z*-score, r = −0.31; FEV_1_/FVC *z*-score, *r* = −0.31; FEF_25-75_ *z*-score, *r* = −0.33), and directly proportional to the percentage change in FEV_1_ after bronchodilator use (*r* = 0.36; *P* < .01 for all comparisons) (Figure). In contrast, in the classmate controls, AIx was correlated only with FEF_25-75_ (*r* = −0.23; *P* < .05) and percent change in FEV_1_ after bronchodilator use (*r* = 0.28; *P* = .01). Pulse pressure amplification was related to FEV_1_/FVC ratio in both EP children (*r* = 0.25; *P* = .04) and their classmates (*r* = 0.25; *P* = .02). MAP, diastolic BP, and systolic BP were not related to lung function measurements in the EP children or classmates.

On multiple regression analysis, in all subjects, AIx was independently and inversely associated with baseline lung function, with a 2.7% increase in AIx for each *z*-score decrease in FEV_1_ (Table IV). There was an additional affect of maternal smoking during pregnancy, associated with a 4.9% increase in AIx. Given that maternal smoke exposure was not recorded in all subjects (Table I), the model was also run without this variable; a similar association between baseline FEV_1_ *z*-score and AIx was observed (data not shown).

Similar findings were observed when FEV_1_/FVC and FEF_25-75_ were substituted for FEV_1_ in the model. In the model including baseline FEV_1_, there was no significant additional contribution of percent change in FEV_1_ after bronchodilator use, because of the strong inverse relationship between these outcomes. However, using percent change in FEV_1_ after bronchodilator use instead of FEV_1_ in the model, a 2.5% increase in AIx for each 5% increase in FEV_1_ after bronchodilator use was seen, with an additional 5.2% increase in AIx if the mother had smoked during pregnancy. EP birth, previous BPD, and current asthma did not contribute further to the model.

In models restricted to EP children alone, baseline FEV_1_ or percent change in FEV_1_ with bronchodilator use had similar effects as in the whole group (Table IV). Although maternal smoking in pregnancy was no longer significant (presumably because of the smaller sample size and thus the lower power of the study during subgroup analysis), it had the same size effect as in the whole group. BPD status had no statistically independent effect on AIx in these models.

## Discussion

We have demonstrated that the increased pulse wave reflection, manifested as an increased AIx value, is independently associated with impaired lung function in children born at 25 weeks of gestation or less compared with term-born classmates at age 11 years. Importantly, these abnormal large-vessel hemodynamics are not reflected in peripheral BP, the standard method of assessing hemodynamics at this age, and no association was seen between lung function and peripheral BP. This novel association is relevant to both pediatric and adult practice as increasing numbers of EP children are reaching adulthood and must be considered at increased risk for later cardiorespiratory morbidity based on these observations.

AIx not only was inversely related to baseline lung function, but also had independent associations with bronchodilator response and maternal smoking during pregnancy. Once these factors had been accounted for, whether or not there is any further detrimental effect of preterm birth over and above that manifested through the poor respiratory function is uncertain. When multiple regression analysis was limited to the children born EP, a striking independent effect of lung function remained evident in this population.

EP birth is followed by significant respiratory and nutritional problems, which lead to poor respiratory health and growth during early childhood.^10,19,20^ Integral to the preterm birth event and the consequent early abrupt changes to fetal oxygenation and hemodynamics are the maternal or fetal factors contributing to the preterm birth, as well as the associated perinatal events such as the use of supplemental oxygen or ventilator-induced lung injury. These factors further complicate the search for specific causal pathways of respiratory and cardiovascular risks. Thus, mechanisms remain speculative, but are known to include a common developmental pathway. Our data clearly demonstrate that we can no longer consider the cardiovascular or respiratory sequelae in isolation if we are to research how to optimize health following preterm birth.

Lung distensibility and elastic recoil are governed primarily by the size of alveoli and surface tension in the healthy lung, but elastin and collagen contribute as well. The alveolar ducts contain a spiral of collagen and elastin, and the interstitium contains bundles of collagen surrounding electron-dense elastin. Within the aorta, elastin contributes 40%-50% of the dry weight in healthy subjects.^37^ Because deposition of elastin within the aorta and in the respiratory tract occurs late in gestation, it may be disrupted after preterm birth and may influence both large arterial wall and lung composition. However, the increase in AIx value associated with increased wave reflection suggests that a structural contribution to the abnormalities might involve the elastic arteries juxtaposing the more muscular resistant peripheral arteries or the microvasculature.

Alterations in angiogenic factors related to preterm birth, such as disruption of vascular endothelial growth factor and vascular/nitric oxide signaling, can lead to structural changes of the cardiorespiratory system.^38,39^ The role of inflammatory changes in the genesis of preterm birth and the evolution of neonatal lung disease and brain injury is a matter of much debate.^40^ Although systemic inflammatory mediators are thought to contribute to the association between reduced lung function and increased arterial stiffness in both healthy and ill adults, there is little causal evidence to confirm this.^4^ It may be hypothesized that lung epithelial injury potentiates a systemic inflammatory state, which in turn leads to endothelial dysfunction or architectural disruption of large arteries.^41^

Maternal smoking during pregnancy increases fetal nicotine exposure and is likely to affect both lung function and arterial stiffness, as passive and direct smoking do in later life.^42^ Increased fetal carboxyhemoglobin leads to hypoxemia and, in murine models, increased oxidative stress when exposed to smoke during the neonatal period.^43^ Supporting this finding are altered hemodynamics in utero and in children whose mothers smoked.^44,45^ Importantly, in this study, the negative impact of maternal smoking on AIx was in addition to that attributed to reduced lung function.

The fetal and perinatal periods are times of rapid growth and development with increased vulnerability to a wide range of insults. Although few insults in the perinatal period compare with that of being born EP, the potential impact of early life exposures for other cardiorespiratory conditions can be illuminating. Despite the clear association between COPD and cumulative tobacco smoke exposure, there is marked heterogeneity of COPD phenotypes and susceptibility, related not only to genetic susceptibility, but also to early life exposures.^46^ Thus, although the altered hemodynamics reported in adults with chronic lung diseases such as COPD and cystic fibrosis have been attributed to an accelerated ageing of the vasculature, these physiological changes may originate in and be triggered by early life factors.

It should be noted that this study is cross-sectional in nature, and any causal relationship can only be inferred. However, the EPICure study provides the opportunity to track cardiorespiratory status and determine future consequences. Further, this work is intended to challenge our current thinking of considering each system in isolation. AIx was based on aortic pressure waveform data, derived from radial pressure recordings, using a generalized transfer function that has not been validated in children. However, McEniery et al^18^ reported similar findings in this group with radial AIx, which does not rely on this transfer function. We did not have detailed data on family history of cardiovascular disease or on cardiovascular status, patent ductus arteriosus, or pulmonary hypertension in infancy. Our findings raise the question of whether a detailed evaluation of neonatal cardiovascular findings, complications, and treatments now requires study of the ontogeny of cardiovascular impairment in EP children and the extent to which treatments for BPD and cardiovascular problems may modify adult outcomes across systems.

In conclusion, the increased AIx values found at age 11 years in EP children are inversely related to baseline lung function and directly related to bronchodilator response, with an additive contribution from exposure to maternal smoking during fetal development. Children born EP who are at increased risk for future cardiovascular and pulmonary problems as adults have alterations suring childhood that are not reflected in traditional measures of peripheral BP.

## Figures and Tables

**Table II:** Comparison of study population and cohort of EP children tested at school at approximately 11 years of age

	EP without hemodynamics (n = 121)	EP with hemodynamics (n = 66)	Mean (95% CI) of difference (b−a)
Male sex, n (%)	48/121 (40)	32/66 (48)	8% (−6% to 22%)
White ethnicity, n/N (%)	92/114 (81)	45/56 (80)	−1% (−14% to 11%)
Gestational age, weeks	25.0 (0.8)	24.9 (0.8)	−0.17 (−0.39 to 0.05)
Birth weight, kg	0.75 (0.11)	0.74 (0.13)	−0.01 (−0.05 to 0.03)
Birth weight *z*-score	−0.15 (0.76)	−0.15 (0.77)	0.01 (−0.23 to 0.24)
BPD, n/N (%)	87/121 (72)	45/66 (68)	−4% (−18% to 9%)
Smoking in pregnancy, n/N (%)	30/113 (27)	13/55 (24)	−3% (−17% to 11%)
Details at time of test			
Age, years	10.9 (0.4)	11.3 (0.5)	0.4 (0.3 to 0.5)∗
Height *z*-score	−0.57 (1.03)	−0.58 (0.97)	−0.01 (−0.31 to 0.30)
Weight *z*-score	−0.60 (1.35)	−0.29 (1.12)	0.32 (−0.07 to 0.70)
Current asthma, n/N (%)	30/117 (26)	14/57 (25)	−1% (−14% to 13%)
FEV_1_*z*-score	−1.54 (1.2)	−1.25 (1.2)	0.30 (−0.06 to 0.66)

Data are mean (SD) unless noted otherwise.

∗ *P* < .01.

**Table III:** Comparison of hemodynamic and lung function values in children born EP, with and without BPD, and control classmates

	EP with BPD (n = 45)	EP, no BPD (n = 21)	All EP (n = 66)	Controls (n = 86)	Mean (95% CI) difference (EP−controls)
Cardiovascular					
Heart rate, bpm	82.5 (11.8)	81.1 (10.6)	82.0 (11.4)	79.6 (11.2)	2.4 (−1.2 to 6.0)
Peripheral systolic BP, mmHg	107.6 (10.9)	109.7 (8.9)	108.2 (10.3)	109.7 (9.2)	−1.5 (−4.6 to 1.6)
Peripheral diastolic BP, mmHg	64.6 (7.3)	65.2 (5.2)	64.8 (6.7)	62.7 (7.1)	2.1 (−0.1 to 4.3)
Peripheral MAP, mmHg	79.4 (7.0)	80.0 (5.6)	79.6 (6.6)	77.6 (6.7)	2.1 (−0.1 to 4.2)
Peripheral pulse pressure, mmHg	43.0 (10.7)	44.5 (7.8)	43.5 (9.8)	47.1 (8.7)	−3.6 (−6.6 to −0.6)∗
Central systolic BP, mmHg	92.3 (7.3)	93.7 (7.5)	92.7 (7.3)	91.4 (7.2)	1.4 (−1.0 to 3.7)
Central diastolic BP, mmHg	66.2 (7.5)	66.6 (5.1)	66.3 (6.8)	64.1 (7.1)	2.2 (−0.1 to 4.4)
Central pulse pressure, mmHg	26.1 (5.5)	27.1 (6.2)	26.5 (5.7)	27.2 (5.4)	−0.8 (−2.6 to 1.0)
AIx, %	8 (9)	7 (11)	7 (10)	2 (9)	5 (2 to 8)†
Aortic pulse wave velocity, m/s	4.5 (0.5)	4.5 (0.6)	4.5 (0.5)	4.7 (0.9)	−0.21 (−0.46 to 0.03)
Pulse pressure amplification	1.65 (0.27)	1.66 (0.15)	1.66 (0.24)	1.74 (0.11)	−0.08 (−0.14 to −0.02)‡
Lung function					
Respiratory rate, breaths/minute	19.1 (3.0)	19.3 (3.0)	19.2 (3.0)	17.7 (2.9)	1.5 (0.5 to 2.4)‡
FEV_1_*z*-score§	−1.54 (1.11)	−0.63 (1.20)	−1.25 (1.21)	−0.23 (1.00)	−1.02 (−1.37 to −0.66)‡
% of predicted	82.1 (12.8)	92.7 (14.1)	85.5 (14.0)	97.3 (11.7)	−11.80 (−15.94 to −7.68)‡
FVC *z*-score§	−0.89 (1.14)	−0.41 (1.16)	−0.74 (1.16)	−0.25 (0.95)	−0.49 (−0.83 to −0.15)‡
% of predicted	90.2 (12.4)	95.5 (13.1)	91.9 (12.8)	97.2 (10.5)	−5.31 (−9.05 to −1.58)‡
FEV_1_/FVC *z*-score§	−1.14 (1.09)	−0.38 (1.30)	−0.90 (1.21)	−0.11 (0.9)	−0.79 (−1.13 to −0.44)‡
FEF_25-75_*z*-score§	−2.04 (1.12)	−1.03 (1.37)	−1.72 (1.29)	−0.53 (1.07)	−1.19 (−1.57 to −0.81)‡
% change in FEV_1_ with bronchodilator use¶,‖	11 (13)	7 (10)	9 (11)	3 (5)	5 (3 to 8)‡
Increase in FEV_1_ >12%, n (%)¶	18 (43%)	3 (18%)	21 (36%)	7 (9%)	27% (13% to 40%)‡
Postbronchodilator FEV_1_*z*-score¶	−0.75 (0.95)	−0.25 (1.14)	−0.61 (1.02)	0.08 (0.95)	−0.69 (−1.02 to −0.36)‡

Data are presented as mean (SD) unless stated otherwise.

∗ *P* < .05 between all EP children and classmates.

† *P* < .001 between all EP children and classmates.

‡ *P* < .01 between all EP children and classmates.

§ Spirometry *z*-scores adjusting for sex, height, and age derived from Stanojevic et al.^36^

¶ Includes 59 EP infants (17 without BPD and 42 with EP) and 79 classmates.

‖ Mann-Whitney test. Results are expressed as median and IQR.

**Figure:**
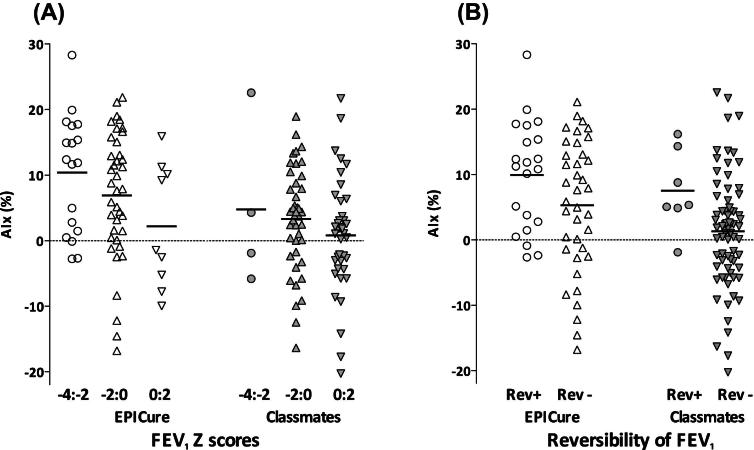
Relationship of **A**, AIx to FEV_1_*z*-score and **B**, reversibility of FEV_1_ (change >12%) in 11-year-old children born at ≤25 weeks gestation (EPICure) and control classmates. *REV*+, significant reversibility; *REV*−, no significant reversibility.

**Table I:** Demographic data of the EP children and classmate controls

	EP with BPD (n = 45)	EP without BPD (n =21)	All EP (n = 66)	Controls (C) (n = 86)	Mean (95% CI) difference (EP−C)
Gestational age, weeks	24.8 (0.8)	25.0 (0.7)	24.9 (0.8)	All >37 weeks	
Birth weight, kg	0.73 (0.13)	0.78 (0.12)	0.74 (0.13)		
Birth weight *z*-score∗	−0.26 (0.69)	0.08 (0.90)	−0.15 (0.77)		
Male sex, n (%)	24 (53)	8 (38)	32 (48)	38 (45)	3% (−13% to 19%)
Smoking in pregnancy n/N (%)	9/36 (23)	4 /19 (21)	13/55 (24)	9/78 (12)	12% (−1% to 25%)
White ethnicity, n/N (%)	27/35 (77)	18 (86)	45/56 (80)	59/69 (86)	−5% (−18% to 8%)
Maternal nonmanual occupation, n/N (%)	18/30 (60)	9/18 (50)	27/48 (56)	42/70 (60)	−4% (−22% to 14%)
Maternal continuing education after 16 years, n/N (%)	15/36 (42)	9/20 (45)	24/56 (42)	37/75 (49)	−6% (24% to 11%)
At time of assessment					
Age, years	11.3 (0.5)	11.3 (0.6)	11.3 (0.5)	11.3 (0.6)	0.0 (−0.2 to 0.2)
Height, cm	141.6 (7.5)	141.3 (6.8)	141.5 (7.2)	147.1 (7.6)	−5.6 (−8.0 to −3.2)†
Height *z*-score∗	−0.55 (1.00)	−0.64 (0.90)	−0.58 (0.97)	0.25 (1.03)	−0.83 (−1.15 to −0.50)‡
Weight, kg	36.3 (7.9)	35.4 (7.0)	36.0 (7.6)	40.0 (9.7)	−4.0 (−6.8 to −1.2)‡
Weight *z*-score∗	−0.24 (1.13)	−0.40 (1.10)	−0.29 (1.12)	0.26 (1.15)	−0.54 (−0.91 to −0.18)‡
Current asthma, n/N (%)	8/37 (22)	6/20 (30)	14/57 (25)	11/ 82 (13)	12% (−1% to 26%)

Data are presented as mean (SD) unless noted otherwise. Current asthma is defined as currently symptomatic and/or with doctor diagnosis of asthma and on medication during the previous 12 months. Not all subjects reported by McEniery et al^27^ with hemodynamic data (68 EP children and 90 controls) had valid spirometry data; thus, the results from 66 children born EP and 86 controls form the basis of this report. Note that accurate gestational age and birth weight data were not available for controls.

∗ Derived from Cole et al.^34^

† *P* < .001 between all EP children and control classmates.

‡ *P* < .01 between all EP children and control classmates.

**Table IV:** Results of stepwise multiple regression analysis with AIx as the dependent variable and including either FEV_1_*z*-score or percent change in FEV_1_ after bronchodilator use

	B	95% CI	Adjusted *r*^2^, %
All subjects			
FEV_1_*z*-score			
Constant	20.6	10.1 to 31.1	
FEV_1_*z*-score	−2.7	−3.9 to −1.5	10.6
Heart rate, bpm	−0.2	−0.4 to −0.1	16.9
Mother smoked during pregnancy	4.9	1.0 to 8.8	20.1
Percent change in FEV_1_ after bronchodilator use			
Constant	15.2	4.0 to 26.4	
FEV_1_% change	0.5	0.3 to 0.7	14.1
Heart rate, bpm	−0.2	−0.31 to −0.1	18.1
Mother smoked during pregnancy	5.2	1.2 to 9.3	21.9
EP children alone∗			
FEV_1_*z*-score†			
Constant	23.7	5.4 to 41.9	
FEV_1_*z*-score	−2.2	−4.2 to −0.2	8.7
Heart rate, bpm	−0.2	−0.4 to −0.01	14.4
Percent change in FEV_1_ after bronchodilator use‡			
Constant	22.0	3.6 to 40.5	
FEV_1_ % change	0.4	0.1 to 0.7	14
Heart rate, bpm	−0.2	−0.4 to −0.01	19.9

*B*, B test; *bpm*, beats per minute.

Variables entered in all models: age at testing (years), MAP (mmHg), heart rate (bpm), height (cm), sex (male/female), BPD status (yes/no), maternal smoking during pregnancy (yes/no), and EP (yes/no).

∗ Although smoking in pregnancy was not significant in these models, it had the same effect size as in the whole cohort.

† B = 5.0; 95% CI, −0.7 to 10.7.

‡ B = 4.8; 95% CI, −1.0 to 10.6.

## Appendix

EPICure Studies Steering Committee Members: Peter Brocklehurst (Chairman), Jane Abbott, Andrew Bush, Richard Cooke, Noreen Maconochie, Alison Matthews, David Matthews, Richard Morton, Maggie Redshaw, David Taylor, Diane Turner, Nigel Turner, and Patrick Walsh.

## References

[1] Hole D.J., Watt G.C.M., Davey-Smith G., Hart C.L., Gillis C.R., Hawthorne V.M. (1996). Impaired lung function and mortality risk in men and women: findings from the Renfrew and Paisley prospective population study. BMJ.

[2] Sin D.D., Man S.F. (2005). Chronic obstructive pulmonary disease as a risk factor for cardiovascular morbidity and mortality. Proc Am Thorac Soc.

[3] Laurent S., Cockcroft J., Van B.L., Boutouyrie P., Giannattasio C., Hayoz D. (2006). Expert consensus document on arterial stiffness: methodological issues and clinical applications. Eur Heart J.

[4] Sabit R., Bolton C.E., Edwards P.H., Pettit R.J., Evans W.D., McEniery C.M. (2007). Arterial stiffness and osteoporosis in chronic obstructive pulmonary disease. Am J Respir Crit Care Med.

[5] Maclay J.D., McAllister D.A., Mills N.L., Paterson F.P., Ludlam C.A., Drost E.M. (2009). Vascular dysfunction in chronic obstructive pulmonary disease. Am J Respir Crit Care Med.

[6] Hull J.H., Garrod R., Ho T.B., Knight R.K., Cockcroft J.R., Shale D.J. (2009). Increased augmentation index in patients with cystic fibrosis. Eur Respir J.

[7] Bolton C.E., Cockcroft J.R., Sabit R., Munnery M., McEniery C.M., Wilkinson I.B. (2009). Lung function in mid-life compared to later life is a stronger predictor of arterial stiffness in men: the Caerphilly Prospective Study (CaPS). Int J Epidemiol.

[8] Lum S., Bush A., Stocks J. (2011). Clinical pulmonary function testing for children with bronchopulmonary dysplasia. Pediatr Allergy Immunol Pulmonol.

[9] Wong P.M., Lees A.N., Louw J., Lee F.Y., French N., Gain K. (2008). Emphysema in young adult survivors of moderate to severe bronchopulmonary dysplasia. Eur Respir J.

[10] Baraldi E., Filippone M. (2007). Chronic lung disease after premature birth. N Engl J Med.

[11] Doyle L.W. (2008). Cardiopulmonary outcomes of extreme prematurity. Semin Perinatol.

[12] Field D.J., Dorling J.S., Manktelow B.N., Draper E.S. (2008). Survival of extremely premature babies in a geographically defined population: prospective cohort study of 1994-9 compared with 2000-5. BMJ.

[13] Filippone M., Sartor M., Zacchello F., Baraldi E. (2003). Flow limitation in infants with bronchopulmonary dysplasia and respiratory function at school age. Lancet.

[14] Sears M.R., Greene J.M., Willan A.R., Wiecek E.M., Taylor D.R., Flannery E.M. (2003). A longitudinal, population-based, cohort study of childhood asthma followed to adulthood. N Engl J Med.

[15] Stern D.A., Morgan W.J., Wright A.L., Guerra S., Martinez F.D. (2007). Poor airway function in early infancy and lung function by age 22 years: a non-selective longitudinal cohort study. Lancet.

[16] Silverman M., Kuehni C.E. (2007). Early lung development and COPD. Lancet.

[17] Nilsson P.M., Lurbe E., Laurent S. (2008). The early life origins of vascular ageing and cardiovascular risk: the EVA syndrome. J Hypertens.

[18] Godfrey K.M., Barker D.J. (2001). Fetal programming and adult health. Public Health Nutr.

[19] Hennessy E.M., Bracewell M., Wood N., Wolke D., Costeloe K., Gibson A. (2008). Respiratory health in pre-school and school age children following extremely preterm birth. Arch Dis Child.

[20] Bracewell M.A., Hennessy E.M., Wolke D., Marlow N. (2008). The EPICure study: growth and blood pressure at 6 years of age following extremely preterm birth. Arch Dis Child Fetal Neonatal Ed.

[21] Lurbe E., Torro M.I., Carvajal E., Alvarez V., Redon J. (2003). Birth weight impacts on wave reflections in children and adolescents. Hypertension.

[22] Oren A., Vos L.E., Bos W.J., Safar M.E., Uiterwaal C.S., Gorissen W.H. (2003). Gestational age and birth weight in relation to aortic stiffness in healthy young adults: two separate mechanisms?. Am J Hypertens.

[23] Tauzin L., Rossi P., Giusano B., Gaudart J., Boussuges A., Fraisse A. (2006). Characteristics of arterial stiffness in very low birth weight premature infants. Pediatr Res.

[24] Broyd C., Harrison E., Raja M., Millasseau S.C., Poston L., Chowienczyk P.J. (2005). Association of pulse waveform characteristics with birth weight in young adults. J Hypertens.

[25] Koudsi A., Oldroyd J., McElduff P., Banerjee M., Vyas A., Cruickshank J.K. (2007). Maternal and neonatal influences on, and reproducibility of, neonatal aortic pulse wave velocity. Hypertension.

[26] Montgomery A.A., Ben Shlomo Y., McCarthy A., Davies D., Elwood P., Smith G.D. (2000). Birth size and arterial compliance in young adults. Lancet.

[27] McEniery C.M., Bolton C.E., Fawke J., Hennessy E., Stocks J., Wilkinson I.B. (2011). Cardiovascular consequences of extreme prematurity: the EPICure Study. J Hypertens.

[28] McEniery C.M., Yasmin, Maki-Petaja K.M., McDonnell B.J., Munnery M., Hickson S.S., Anglo-Cardiff Collaboration Trial Investigators (2010). The impact of cardiovascular risk factors on aortic stiffness and wave reflections depends on age: the Anglo-Cardiff Collaborative Trial (ACCT III). Hypertension.

[29] Fawke J., Lum S., Kirkby J., Hennessy E., Marlow N., Rowell V. (2010). Lung function and respiratory symptoms at 11 years in children born extremely preterm: the EPICure study. Am J Respir Crit Care Med.

[30] Kirkby J., Welsh L., Lum S., Fawke J., Rowell V., Thomas S. (2008). The EPICure study: comparison of pediatric spirometry in community and laboratory settings. Pediatr Pulmonol.

[31] Lum S., Kirkby J., Welsh L., Marlow N., Hennessy E., Stocks J. (2011). Nature and severity of lung function abnormalities in extremely preterm children at 11 y. Eur Respir J.

[32] Walker S.M., Franck L.S., Fitzgerald M., Myles J., Stocks J., Marlow N. (2009). Long-term impact of neonatal intensive care and surgery on somatosensory perception in children born extremely preterm. Pain.

[33] Johnson S., Hollis C., Kochhar P., Hennessy E., Wolke D., Marlow N. (2010). Psychiatric disorders in extremely preterm children: longitudinal finding at age 11 years in the EPICure study. J Am Acad Child Adolesc Psychiatry.

[34] Cole T.J., Freeman J.V., Preece M.A. (1998). British 1990 growth reference centiles for weight, height, body mass index and head circumference fitted by maximum penalized likelihood. Stat Med.

[35] Pauca A.L., O'Rourke M.F., Kon N.D. (2001). Prospective evaluation of a method for estimating ascending aortic pressure from the radial artery pressure waveform. Hypertension.

[36] Stanojevic S., Wade A., Cole T.J., Lum S., Custovic A., Silverman M., Asthma UK Spirometry Collaborative Group (2009). Spirometry centile charts for young Caucasian children: the Asthma UK Collaborative Initiative. Am J Respir Crit Care Med.

[37] Martyn C.N., Greenwald S.E. (1997). Impaired synthesis of elastin in walls of aorta and large conduit arteries during early development as an initiating event in pathogenesis of systemic hypertension. Lancet.

[38] Lassus P., Turanlahti M., Heikkilä P., Andersson L.C., Nupponen I., Sarnesto A. (2001). Pulmonary vascular endothelial growth factor and Flt-1 in fetuses, in acute and chronic lung disease, and in persistent pulmonary hypertension of the newborn. Am J Respir Crit Care Med.

[39] Fujinaga H., Baker C.D., Ryan S.L., Markham N.E., Seedorf G.J., Balasubramaniam V. (2009). Hyperoxia disrupts vascular endothelial growth factor–nitric oxide signaling and decreases growth of endothelial colony-forming cells from preterm infants. Am J Physiol Lung Cell Mol Physiol.

[40] O'Reilly M.A., Marr S.H., Yee M., Grath-Morrow S.A., Lawrence B.P. (2008). Neonatal hyperoxia enhances the inflammatory response in adult mice infected with influenza A virus. Am J Respir Crit Care Med.

[41] Roman M.J., Devereux R.B., Schwartz J.E., Lochshin M.D., Paget S.A., Davis A. (2005). Arterial stiffness in chronic inflammatory diseases. Hypertension.

[42] Mahmud A., Feely J. (2003). Effect of smoking on arterial stiffness and pulse pressure amplification. Hypertension.

[43] McGrath-Morrow S., Rangasamy T., Cho C., Sussan T., Neptune E., Wise R. (2008). Impaired lung homeostasis in neonatal mice exposed to cigarette smoke. Am J Respir Cell Mol Biol.

[44] Lawlor D.A., Najman J.M., Sterne J., Williams G.M., Ebrahim S., Davey S.G. (2004). Associations of parental, birth, and early life characteristics with systolic blood pressure at 5 years of age: findings from the Mater University study of pregnancy and its outcomes. Circulation.

[45] Kyrklund-Blomberg N.B., Hu J., Gennser G. (2006). Chronic effects of maternal smoking on pulse waves in the fetal aorta. J Matern Fetal Neonatal Med.

[46] de Marco R., Accordini S., Marcon A., Cerveri I., Antó J.M., Gisalason T., European Community Respiratory Health Survey (ECRHS) (2011). Risk factors for chronic obstructive pulmonary disease in a European cohort of young adults. Am J Respir Crit Care Med.

